# Dietary bile acid supplementation improves the intestinal health and growth performance of piglets partially through the FXR/AQPs pathway

**DOI:** 10.1186/s40813-025-00440-x

**Published:** 2025-05-21

**Authors:** Beibei Zhang, Min Tian, Yahui Yang, Yueqin Qiu, Li Wang, Hao Xiao, Xiaoping Zhu, Limei Qin, Xuefen Yang, Zongyong Jiang

**Affiliations:** 1 Institute of Animal Science, Guangdong Academy of Agricultural Sciences, Guangdong, China; 2 State Key Laboratory of Swine and Poultry Breeding Industry, Guangdong, China; 3 Key Laboratory of Animal Nutrition and Feed Science in South China, Ministry of Agriculture and Rural Affairs, Guangdong, China; 4 Guangdong Provincial Key Laboratory of Animal Breeding and Nutrition, Guangdong, China; 5 School of Life Sciences and Engineering, Foshan University, Foshan, China

**Keywords:** Bile acid, Piglet, Growth performance, Gut health, FXR

## Abstract

**Background:**

Maintaining the integrity of the structure and function of piglet intestines is crucial for their growth and health. This study aims to evaluate the effects of an antibiotic free diet supplemented with bile acid on gut health and growth performance of weaned piglets, and to explore their regulatory mechanisms.

**Methods:**

Thirty-two weaned piglets were randomly divided into two groups and fed either a basal diet or a basal diet supplemented with 350 mg/kg bile acid.

**Results:**

Dietary supplementation with bile acid increased the average daily gain (ADG) and final weight of piglets, and reduced the diarrhea incidence (*P* < 0.05), which was verified to be related to the improvement of lipid absorption, amino acid transport, and intestinal barrier function. Bile acid increased the concentration of lipase and decreased the concentration of total cholesterol, total glyceride, low-density lipoprotein, and urea nitrogen in serum (*P* < 0.05). Meanwhile, bile acid improved the mRNA expression of amino acid transporters in the intestine. On the other hand, bile acid decreased the pH values of the stomach, jejunum, and colon, and improved intestinal morphology (*P* < 0.05). The real-time quantitative PCR results showed that bile acid increased the mRNA expression of Occludin and ZO-1 in the duodenum and ileum (*P* < 0.05). Moreover, dietary bile acid supplementation altered the composition of the ileal microbiota in piglets and increased the relative abundance of *Ligilactobacillus*. In vitro, bile acid improved the repair of IPEC-J2 cells after injury and was shown to be associated with the activation of farnesoid X receptors (FXR) and increased expression of tight junction proteins and aquaporins (AQPs) proteins.

**Conclusion:**

This study found that dietary bile acid supplementation promotes the intestinal health and nutrient absorption partially through the FXR/AQPs pathway, ultimately improving growth performance of piglets.

**Supplementary Information:**

The online version contains supplementary material available at 10.1186/s40813-025-00440-x.

## Introduction

Early weaned piglets are prone to oxidative stress due to incomplete development of the liver-gut system and low immunity, which is mainly manifested by impaired growth performance and diarrhea [1]. The disruption of the intestinal integrity is one of the important factors leading to diarrhea and growth impairment of piglets after weaning [2, 3]. In production, rapid changes in diet and invasion of pathogenic microorganisms are the main factors that disrupt the intestinal function of weaned piglets, as which can easily induce acute inflammation of the piglet intestine [4–6]. Excessive production of pro-inflammatory cytokines in intestinal tissue disrupts tight junctions and increased paracellular permeability [7]. In the past, antibiotics were widely used in the pig industry due to their good disease prevention effect and low price [8]. However, the misuse of antibiotics in animal production and residues in foods have raised awareness of the distinct possibility that their misuse will ultimately threaten human safety [9]. Currently, antibiotic prohibition has become a basic requirement for animal feed production, which has led to an urgent need for effective antibiotic substitutes in animal production.

Bile acid is a green, safe, and efficient new type of feed additive that has received increasing attention in recent years. As is well known, bile acid is the main component of bile and one of the final products of cholesterol metabolism through the liver [10]. The main types of bile acid include chenodeoxycholic acid (CDCA), deoxycholic acid, and cholic acid [11]. CDCA is a main primary bile acid that naturally exists in human and animal bile [12]. Differently, deoxycholic acid is a major secondary bile acid, which can be produced by the conversion of CDCA [13]. CDCA has been reported to have positive effects in improving lipid metabolism and treating cholestasis and biliary tract disorders [10, 14]. In addition to its role in fat metabolism, increasing evidence suggested that bile acid can serve as key signaling molecules, regulating various biological effects by activating specific bile acid receptors and cellular signaling pathways [15, 16]. In the liver and intestines, the nuclear Farnese X receptor (FXR) and membrane G protein coupled receptor TGR5 are the two main sensing receptors for bile acid [16]. Research has shown that FXR not only played an important role in the regulation of the bile acid synthesis, lipid metabolism, and glucose homeostasis, but also was related to the integrity of animal intestinal barrier and immunity [17].

Recently, accumulating evidence suggested that bile acid showed a positive effect on improving the growth and health of pigs [18, 19]. Song et al. (2021) found that dietary supplementation with 200 mg/kg CDCA improved the growth performance of piglet, which was associated with the repair of piglet intestinal integrity. However, further evidence is still needed to clarify its regulatory mechanism. Therefore, this study investigated the effects of dietary supplementation with bile acid on the growth performance and gut health of weaned piglets. Subsequently, this study selected CDCA as the representative component of bile acid and explored the potential mechanism of bile acid improving piglet growth performance and intestinal health based on the porcine small intestinal epithelial cell model, aiming to provide reference for the application of bile acid in pig production.

## Methods and materials

### Ethical statement

All animal protocols used in this study were approved by the Animal Care and Use Committee of the Guangdong Academy of Agricultural Sciences (No. GAASIAS-2016-017, Guangzhou, China).

### Animal experiment

A total of thirty-two male-weaned piglets (21 days old, Duroc × Landrace × Yorkshire) with an average body weight of 7.2 ± 0.38 kg were selected and randomly divided into two groups with eight pens per group and two piglets per pen, fed with either a basal diet (CON group) or a basal diet supplemented with 350 mg/kg bile acid (BA group), and there was no transition period. The trial period is 14 days in total. The determination of bile acid dosage in the experiment referred to previous research reports [18]. The basal diet was formulated according to the National Research Council (NRC, 2012) for piglets at the 7–11 kg phase, and its ingredient composition and nutrient levels are shown in Table 1. No antibiotics are added to the basic diet. The bile acid used in animal experiment contains 70.67% deoxycholic acid, 19.61% CDCA, and 8.00% cholic acid. All piglets were provided *ad libitum* access to feed and water during the experiment.

**Table 1:** Ingredient composition and nutrient levels of the basal diet

Ingredient		Nutrient levels^2^	
Corn	38.07	DE, kcal/kg	3700
Fermented soybean meal	10.00	Total CP, %	22.90
De-hulled soybean meal	11.00	SID Lys, %	1.49
Fishmeal	5.37	SID Met, %	0.52
Whey protein concentrate	5.00	SID Met + Cys, %	0.80
Low protein whey powder	15.00	SID Thr, %	0.87
Sucrose	2.00	SID Trp, %	0.25
Soybean oil	5.00	STTD P, %	0.49
L-Lysine HCl	0.28		
DL-Methionine	0.16		
L-Threonine	0.10		
Calcium hydrophosphate	1.60		
NaCl	0.55		
Choline chloride 50%	0.20		
Wheat shorts	2.67		
Premix^1^	3.00		
Total	100.00		

^1^ Supplied per kilogram of complete diet: Fe, 120 mg; Cu, 10 mg; Zn, 120 mg; Mn, 35 mg; I, 0.25 mg, Se, 0.2 mg; vitamin A, 8000 IU; vitamin D_3_, 1000 IU; vitamin E, 30 mg; vitamin K_3_, 2 mg; vitamin B_1_, 2 mg; vitamin B_2_, 6 mg; vitamin B_6_, 4.0 mg; vitamin B_12_, 0.02 mg; niacin, 25 mg; calcium pantothenate, 10 mg; folic acid, 1.0 mg; biotin, 0.25 mg

^2^ Nutrient levels are calculated according to the NRC (2012) database

The feed intake and diarrhea of piglets were recorded daily. The fecal score and diarrhea rate were statistically analyzed based on the research of Ren et al. (2020). Piglets were weighed at the beginning and end of the experiment after fasted for 12 h. At the end of the experiment, one piglet from each pen was randomly selected to collect blood through the anterior vena cava. Serum was obtained by centrifuging blood at 4 °C and 3000×g. On the morning (8:00 am) of the 15th day of the experiment, piglets were euthanized by injection of pentobarbital sodium (10 mg/kg) after weighing. The pH of the gastrointestinal tract was measured using an electronic pH meter. The liver samples of piglets were collected from the same part of the liver, frozen in liquid nitrogen, and stored in a -80 ℃ freezer until analysis. The intestinal tissue samples of the middle duodenum (sample collected 10 cm after pylorus), middle jejunum (taken at 50 cm after the pylorus), and distal ileum (10 cm before the ileocecal junction) of piglets were collected referring to the methods used in previous research [21]. After washing with physiological saline, intestinal mucosal samples were collected with glass slides. Among them, the samples used for intestinal morphology analysis were fixed in a 4% paraformaldehyde solution; the intestinal mucosal samples were collected in a cryopreserved tube and stored in a -80 °C refrigerator. In addition, samples of ileal contents were collected for sequencing analysis.

### Cell culture experiment

In vitro experiments, the porcine small intestine epithelial cell model (IPEC-J2) was selected for mechanistic studies. IPEC-J2 were cultured in the DMEM/F12 medium supplemented with 10% of fetal bovine serum (FBS), 1% of penicillin and streptomycin in humidified atmosphere with 5% CO_2_ at 37 °C. The medium was changed every 24 h. IPEC-J2 cells were inoculated into 6-well plates with a density of 1 × 10^6^ cells. Once the cells were cultured reaching 60–70% confluence, the cells were cultured with DMEM/F12 medium supplemented with cholic acid.

Before conducting the formal experiment, a preliminary experiment was conducted to compare the effects of different bile acid components on IPEC-J2, including CDCA, deoxycholic acid, and cholic acid. The results showed that 0.5 µmol/L CDCA had the best improvement effect on the viability of IPEC-J2 cells. Therefore, 0.5 µmol/L CDCA was selected for subsequent experiments in this study to validate the mechanism of bile acid regulation of piglet intestinal health. CDCA (Purity > 98%, Sigma) was dissolved in dimethyl sulfoxide (DMSO) for in vitro experiments. Next, the IPEC-J2 cells were stimulated with the Enterotoxigenic *Escherichia coli* (ETEC) K88 (1 × 10^8^ CFU/well) for 4 h. ETEC K88 (CVCC225, toxins LT and ST) was preserved by the Porcine Nutrition Research Group, Institute of Animal Science, Guangdong Academy of Agricultural Sciences, with serotype O149: K91: K88ac, hereinafter referred to as K88. After incubation at 37 °C with shaking for 12 h, bacteria were harvested by centrifugation at 3000×g for 10 min at 4 °C, washed twice with phosphate buffer solution (PBS) and resuspended in DMEM/F12 medium.

To verify the functionality of FXR, FXR siRNAs (Ribobio, Guangzhou, China) were transfected into the IPEC-J2 cells. The empty plasmid vector was used as a negative control. Before the start of the experiment, the silencing efficiency of FXR siRNA was identified through the quantitative real-time PCR.

### Intestinal morphology analysis

According to the standard paraffin embedding technique, the fixed intestinal samples were embedded in paraffin, sliced, and then stained with hematoxylin and eosin staining solution for histopathological evaluation. The entire histological section was scanned using a digital microscope scanner (Pannoramic 250, 3D HISTECH). At least 5 microscopic fields per sample were randomly selected to measure villus height (V) and crypt depth (C) of duodenum, jejunum and ileum by using the Program Image-pro Plus 6.0, and the V/C ratio was calculated.

### Serum biochemical indexes determination

The concentrations of blood urea nitrogen, total cholesterol, triglyceride, high-density lipoprotein (HDL) and low-density lipoprotein (LDL) in serum were determined using the kits purchased from Nanjing Jiancheng Institute of Bioengineering.

### Cell viability analysis

IPEC-J2 cells were cultured in 96-well plates with 3 × 10^5^ cells/well and incubated with CCK8 (Beyotime, Shanghai, China) for 3 h at 24 h after treatment, respectively. Cell viability was evaluated assessed by measuring the OD value in a microplate reader (Multiskan FC, Thermo-Fisher, Rockford, USA) at 450 nm.

### Physical scratch analysis of IPEC-J2 cells

To evaluate the ability of bile acids to improve the intestinal barrier, this study used CDCA as a representative bile acid component and analyzed its ability to repair damaged cells. A 200 µL pipette tip was used to linearly scratch the well differentiated IPEC-J2 cells in 6-well cell culture plates. Then were washed away these scratched cells with PBS 3 times. The cell confluency in 6-well plates was imaged with microscopy every 12 h, and relative wound closure ratios were analyzed using the Program Image-pro Plus 6.0.

### Quantitative real-time PCR

A total of 100 mg intestinal mucosa and liver tissue was added into 0.9 mL of Trizol reagent (Invitrogen, Carlsbad, CA) and homogenized using a low-temperature homogenizer. Then, the supernatant was separated from the homogenate by centrifugation at 12,000 rpm at 4 °C for 5 min. Total RNA was extracted from intestinal mucosa and liver tissue according to the manufacturer’s instructions. For IPEC-J2 cells, 0.5 mL of Trizol per well was added to the cell culture plate after cleaning with phosphate buffer. After the cells shed, a mixture of cells and Trizol was collected for RNA extraction. Next, RNA concentration and quality were determined by a NanoDrop ND-1000 spectrophotometer. Subsequently, first-strand cDNA was synthesized using the Takara reverse transcription kit (Takara, Tokyo, Japan). Bio-Rad quantitative PCR instrument (C1000 Touch, Bio-Rad, USA) and SYBR green (Bio-Rad, USA) were used to detect target genes expression, including lipid metabolism, amino acid transport, tight junction proteins, and aquaporins. All primers were designed using the Primer 5.0 software (Premier Bio-soft International, CA, USA) and synthesized by Sangon Biotech Co. Ltd (Shanghai, China). The primer sequences are shown in Table S1. Samples were normalized to β-actin, and the relative changes of target gene expression were analyzed by the 2^−ΔΔCt^ method.

### Western blot analysis

Total protein from the IPEC-J2 cell was extracted using a Radio Immunoprecipitation Assay Lysis buffer (RIPA) buffer containing a cocktail of protease inhibitors (Biosharp Life sciences, Anhui, China). After centrifugation at 13,000×g for 10 min at 4 °C, the protein concentration in the supernatant fluid was determined using a BCA Protein Assay Kit (Beyotime, Shanghai, China). Subsequently, the total protein separated was prepared into a protein loading buffer. The sample buffer containing 25 µg of total protein was put into a 10% polyacrylamide gel for electrophoresis to separate the target protein. Then, the target protein on the gel was transferred to polyvinylidene difluoride membranes (Millipore, Bedford, MA) and blocked with 5% defatted milk powder. After blocked, the target protein was gradually immersed in a buffer containing a primary or secondary antibody to bind to the antibody. Anti-aquaporins (AQPs) 3 antibodies (1:2000, LifeSpan, USA), anti-FXR (1:2000, Cell Signaling Technology, USA) and anti-β-Actin (Beyotime, Shanghai, China) were used in this study. Finally, the fluorescence signal of the target protein was excited by ECL chemiluminescence solution and quantified by the ChemiDoc XRS imaging system (Thermo, USA). The intensity of bands was analyzed and quantified by Image J Sofware (USA).

### 16 S rDNA sequencing analysis and bioinformatics of bacteria

Microbial genomic DNA from ileal contents was extracted by using the Cetyltrimethylammonium bromide (CTAB) method at Novogene Bioinformatics Technology Co., Ltd., and the DNA quality was analyzed using agarose gel electrophoresis. The V3−V4 region of the 16 S rRNA gene was amplified using universal primers, including the forward primer 341 F (5′-ACTCCTACGGGAGGCAGCA-3′) and reverse primer 806R(5′-TCGGACTACHVGGGTWTCTAAT-3′). Sequencing analyses of 16 S rRNA were based on amplicon sequence variants (ASVs) and were conducted on the Thermo Fisher Ion SSTM XL platform (Novogene, Beijing, China). Low-quality bases from all raw reads were clipped using Cutadapt (version 1.9.1), and the reads were subsequently allocated to distinct samples according to barcode sequences. Then, operational taxonomic units (OTU) clustering was executed by Uparse software (version 7.0.1001), and using USEARCH software (version 10) to define OTUs as a 97% sequence identification threshold. The alpha diversity was analyzed by Shannon’s index and Chao l index. The beta diversity was analyzed using principal component analysis (PCoA) and principal coordinate analysis on unweighted UniFrac distance matrices. Finally, Tax4fun was used to predict the differential metabolic pathways. Additionally, the analysis of the Spearman correlation was done between the expression of genes related to fat metabolism, amino acid transporter, and intestine differential flora.

### Statistical analysis

In animal experiment, the concentration of dietary bile acids was specified as a fixed effect, and the growth performance, feed/gain ratio, diarrhea rates, serum biochemical indicators, mRNA expression of target genes, intestinal morphology, gastrointestinal pH, and gut microbiota of piglets were specified as random effects. In cell culture experiment, the concentration of CDCA and ETEC K88 addition was specified as a fixed effect, and the damage repair ability, cell viability, the expression of FXR, tight junctions, and aquaporins in IPEC-J2 cells were specified as random effects. Data were analyzed using GraphPad Prism version 8.0 (GraphPad Software, USA) and results presented as the means ± standard error of mean (SEM). A one-way ANOVA was performed on multiple groups, followed by Tukey’s multiple comparison test. 0.01 < *P* ≤ 0.05 indicates statistically significant differences (*), *P* ≤ 0.01 indicates a statistically very significant difference (**). The correlations between amino acid transporter and fat metabolism gene expression and intestinal differential flora were assessed using Spearman’s correlation.

## Results

### Growth performance and diarrhea incidence of piglets

The growth performance and diarrhea incidence of piglets are shown in Table 2. At the end of the experiment, the final body weight (BW) and average daily gain (ADG) of piglets in the BA group were significantly higher than those in the CON group (*P* < 0.05). Moreover, dietary supplementation with BA showed a tendency to alleviate diarrhea (*P* = 0.06) and reduce F/G (*P* = 0.07).

**Table 2:** Effects of dietary BA supplementation on growth performance and diarrhea incidence of piglets

Item	CON	BA	SEM	*P*-value
N.	8	8		
Initial BW, kg	7.21	7.24	0.38	0.51
Final BW, kg	12.78	13.49	0.62	0.04
ADG, g/d	398.96	446.37	39.02	0.02
ADFI, g/d	466.71	464.15	29.26	0.14
F/G	1.16	1.04	0.06	0.07
Diarrhea incidence, %	18.75	4.38	5.41	0.06

CON, control group; BA, bile acid group. BW, body weight; ADG, average daily gain; ADFI, average daily feed intake; F/G, feed/gain ratio. Data are presented as the mean ± SEM

### Concentration of serum biochemical indicators

**Fig. 1:**
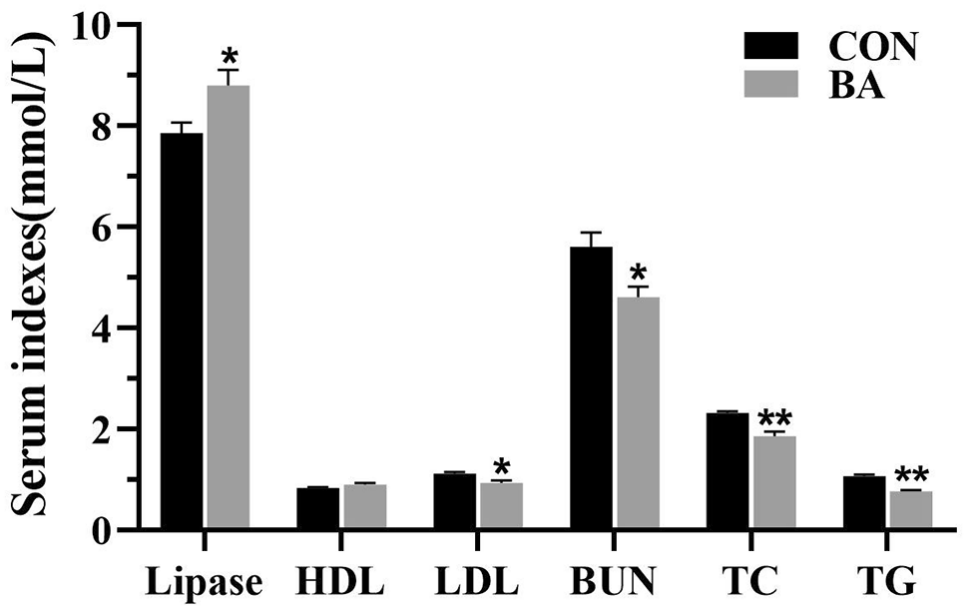
Concentration of biochemical indicators in the serum of piglets. BUN, blood urea nitrogen; TC, total cholesterol; TG, triglyceride; HDL, high-density lipoprotein; LDL, Low-density lipoprotein. Values are presented as the mean ± SEM (*n* = 8). *, 0.01 < *P* ≤ 0.05. **, *P* ≤ 0.01

The concentrations of lipase, blood urea nitrogen, total cholesterol, triglyceride, HDL, and LDL in the serum of piglets are shown in Fig. 1. The concentration of lipase in the serum of piglets in the BA group was significantly higher than that in the CON group (*P* < 0.05). In addition, the serum concentrations of blood urea nitrogen, total cholesterol, triglyceride, and LDL in the BA group were significantly lower than those in the CON group (*P* < 0.05).

### mRNA expression of genes related to fat metabolism in the liver and intestine

**Fig. 2:**
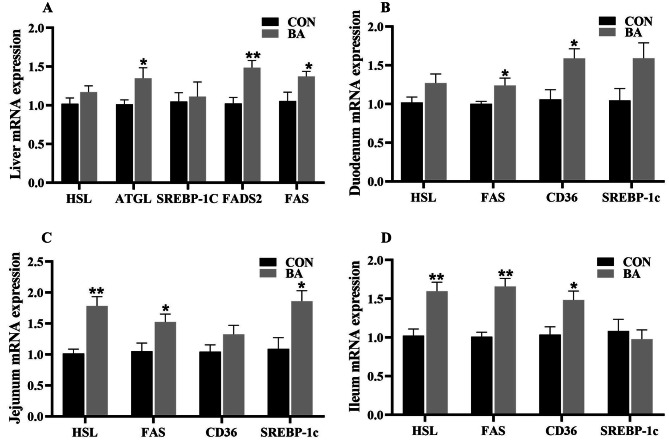
The mRNA expression of key genes related to fat metabolism in the liver and intestinal mucosa of piglets. *FADS2*, recombinant fatty acid desaturase 2; *HSL*, hormone-sensitive triglayceride lipase; *ATGL*, adipose triglyceride lipase; *FAS*, Fatty acid synthase; *CD36*, fatty acid translocase; *SREBP-1c*, sterol regulatory element binding protein-1c. *n* = 8. *, 0.01 < *P* ≤ 0.05. **, *P* ≤ 0.01

In the liver (Fig. 2A), the relative mRNA expression of adipose triglyceride lipase (*ATGL*), recombinant fatty acid desaturase 2 (*FADS2*), and fatty acid synthase (*FAS*) in the BA group were significantly higher than those in the CON group (*P* < 0.05).

In the duodenum (Fig. 2B), the relative mRNA expression of *FAS* and fatty acid translocase (*CD36*) in the BA group were significantly higher than those in the CON group (*P* < 0.05). In the jejunum (Fig. 2C), the relative mRNA expression of hormone-sensitive triglayceride lipase (*HSL*), FAS, and sterol regulatory element binding protein-1c (*SREBP-1c*) in the BA group were significantly higher than those in the CON group (*P* < 0.05). In the ileum (Fig. 2D), the relative mRNA expression of *HSL*, *FAS*, and *CD36* in the BA group were significantly higher than those in the CON group (*P* < 0.05).

### mRNA expression of amino acid transporters in the intestine

**Fig. 3:**
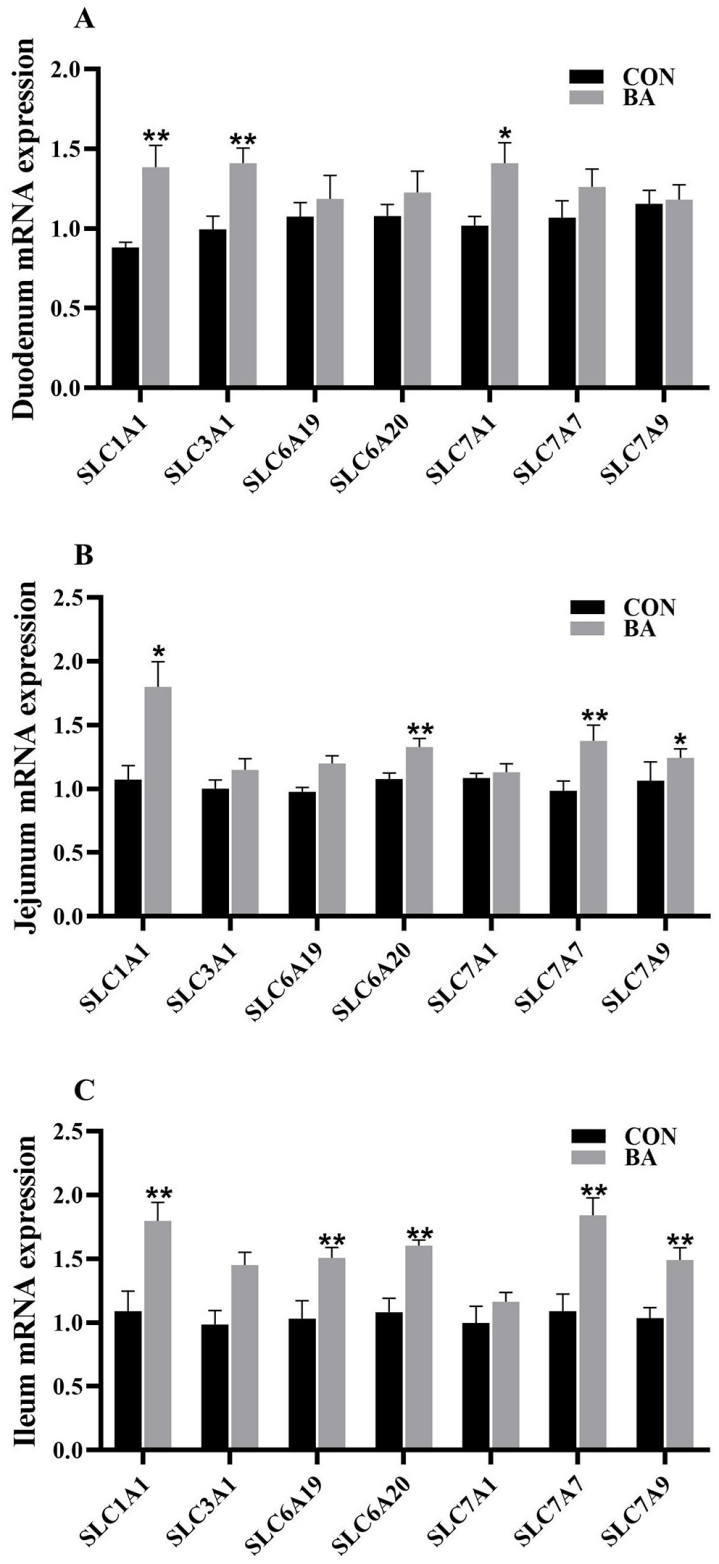
The relative mRNA expression of amino acid transporters in the intestinal mucosa of piglets. solute carrier family 1 member 1 (*SLC1A1*); solute carrier family 3 member 1 (*SLC3A1*); solute carrier family 6 member 19 and 20 (*SLC6A19* and *SLC6A20*); solute carrier family 7 member 1, 7 and 9 (*SLC7A1*, *SLC7A7* and *SLC7A9*). A, duodenum; B, jejunum; C, ileum. *n* = 8. *, 0.01 < *P* ≤ 0.05. **, *P* ≤ 0.01

In the duodenum (Fig. 3A), the amino acid transporter related gene’s mRNA expression of solute carrier family 1 member 1 (*SLC1A1*), solute carrier family 3 member 1 (*SCL3A1*), and solute carrier family 7 member 1 (*SLC7A1*) in the BA group were significantly higher than those in the CON group (*P* < 0.05). In the jejunum (Fig. 3B), the amino acid transporter related gene’s mRNA expression of *SLC1A1*, solute carrier family 6 member 20 (*SLC6A20*), and solute carrier family 7 member 7 and 9 (*SLC7A7* and *SLC7A9*) in the BA group were significantly higher than those in the CON group (*P* < 0.05). In the ileum (Fig. 3C), the amino acid transporter related gene’s mRNA expression of *SLC1A1*, *SLC6A19*, *SLC6A20*, and solute carrier family 7 member 7 and 9 (*SLC7A7* and *SLC7A9*) in the BA group were significantly higher than those in the CON group (*P* < 0.05).

### Gastrointestinal pH

**Fig. 4:**
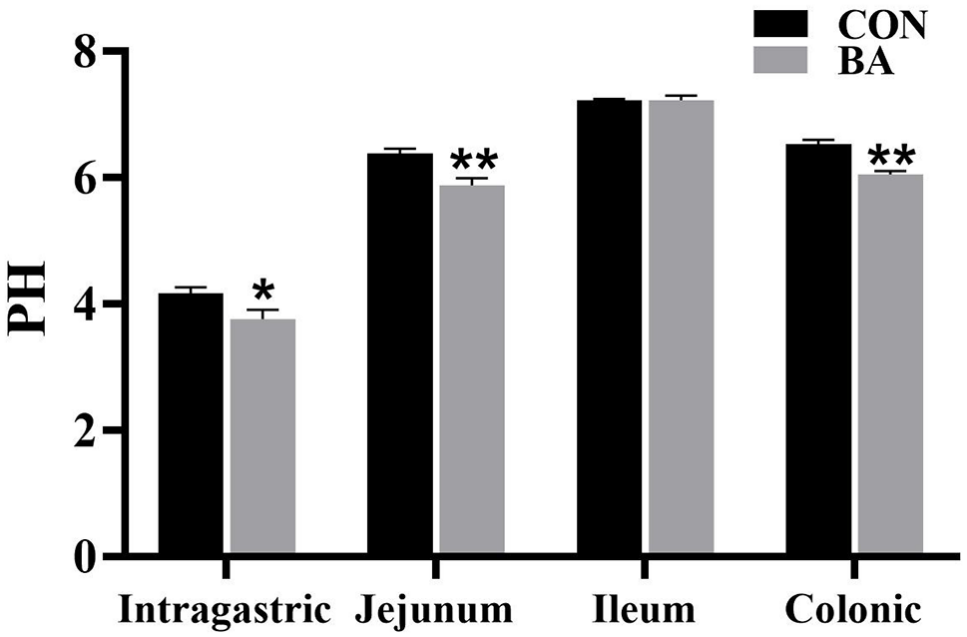
The pH value of the gastrointestinal tract in piglets. *, 0.01 < *P* ≤ 0.05. **, *P* ≤ 0.01

The pH values of the stomach, jejunum, ileum, and colon of piglets are shown in Fig. 4. The pH values of the intragastric, jejunum, and colon in the BA group were significantly lower than those of the CON group (*P* < 0.05).

### Intestinal morphology

**Fig. 5:**
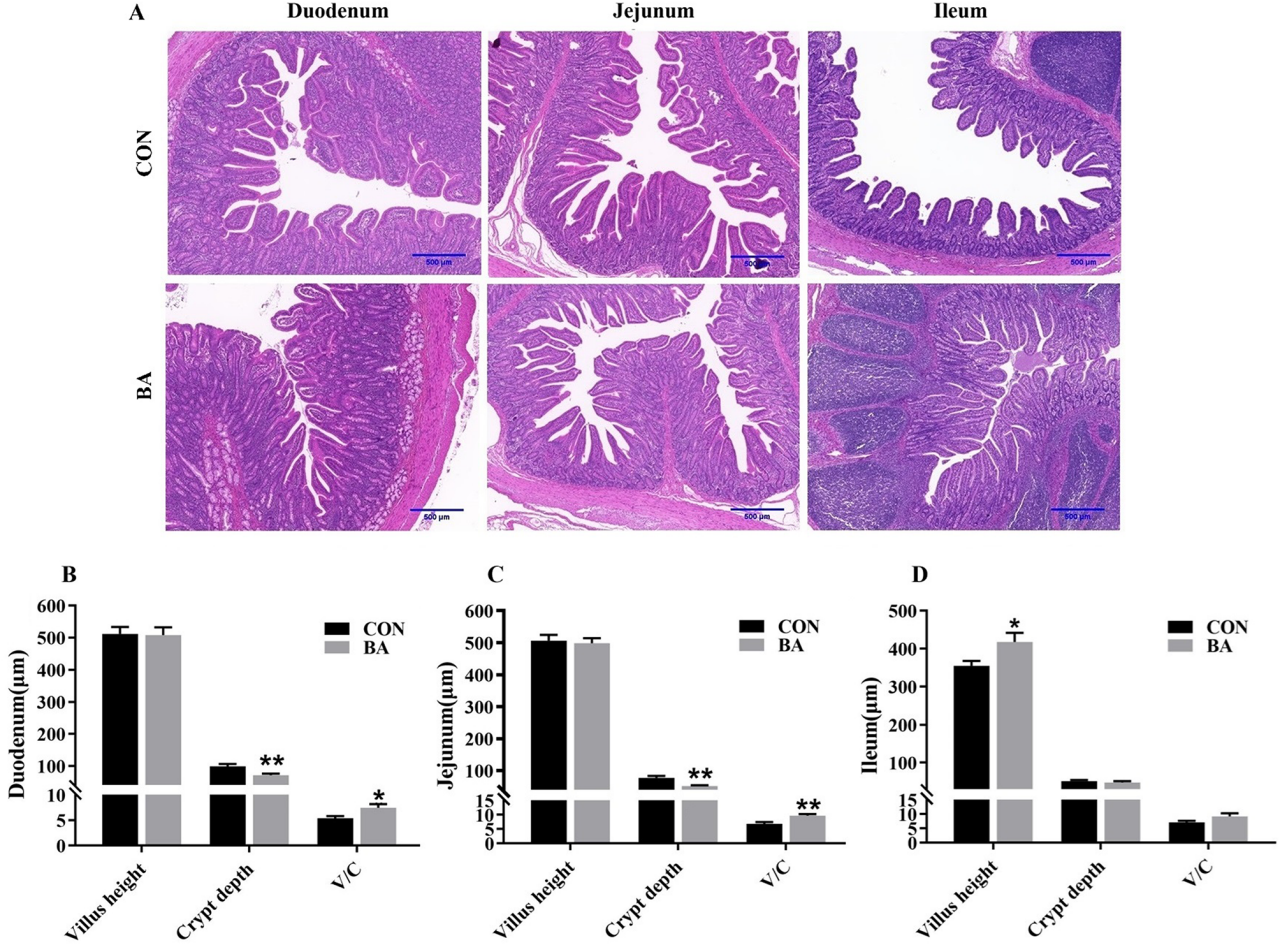
Bile acid supplementation on intestinal morphology of piglets (*n* = 8). **A**, H&E staining of the intestine (scale bar, 500 μm). **B**-**D**, Analysis of villi height and crypt depth in the intestine. Values are presented as the mean ± SEM (*n* = 8). *, 0.01 < *P* ≤ 0.05. **, *P* ≤ 0.01

The intestinal morphology, villus height, and crypt depth of piglets are shown in Fig. 5. In the duodenum and jejunum (Fig. 5B-C), the crypt depth in the BA group was significantly lower than that in the CON group (*P* < 0.05), while the villus height was not significantly different from that in the CON group, which resulted in a significantly higher value of C/V in the BA group compared to the CON group (*P* < 0.05). In the ileum (Fig. 5D), the villus height and C/V value of the BA group were significantly higher than those of the CON group (*P* < 0.05).

### mRNA expression of the tight junctions in the intestine

**Fig. 6:**
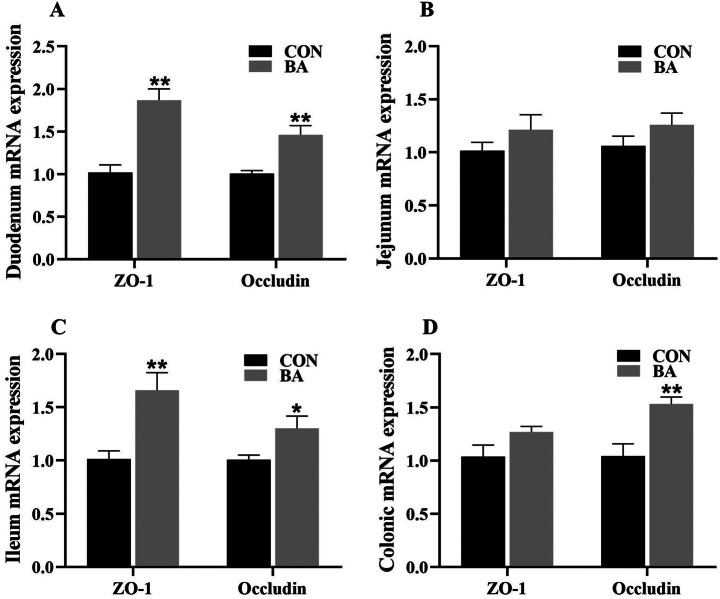
The mRNA expression of tight junctions in the intestine of piglets. BA, bile acid group. Values are presented as the mean ± SEM (*n* = 8). *, 0.01 < *P* ≤ 0.05. **, *P* ≤ 0.01

In the duodenum and ileum (Fig. 6), the mRNA expression of ZO-1 and Occludin in the BA group was significantly higher than that in the CON group (*P* < 0.05). Furthermore, the relative mRNA expression of Occludin in the colon of piglets in the BA group was significantly higher than that in the CON group (*P* < 0.05).

### mRNA expression of AQPs in the intestine

**Fig. 7:**
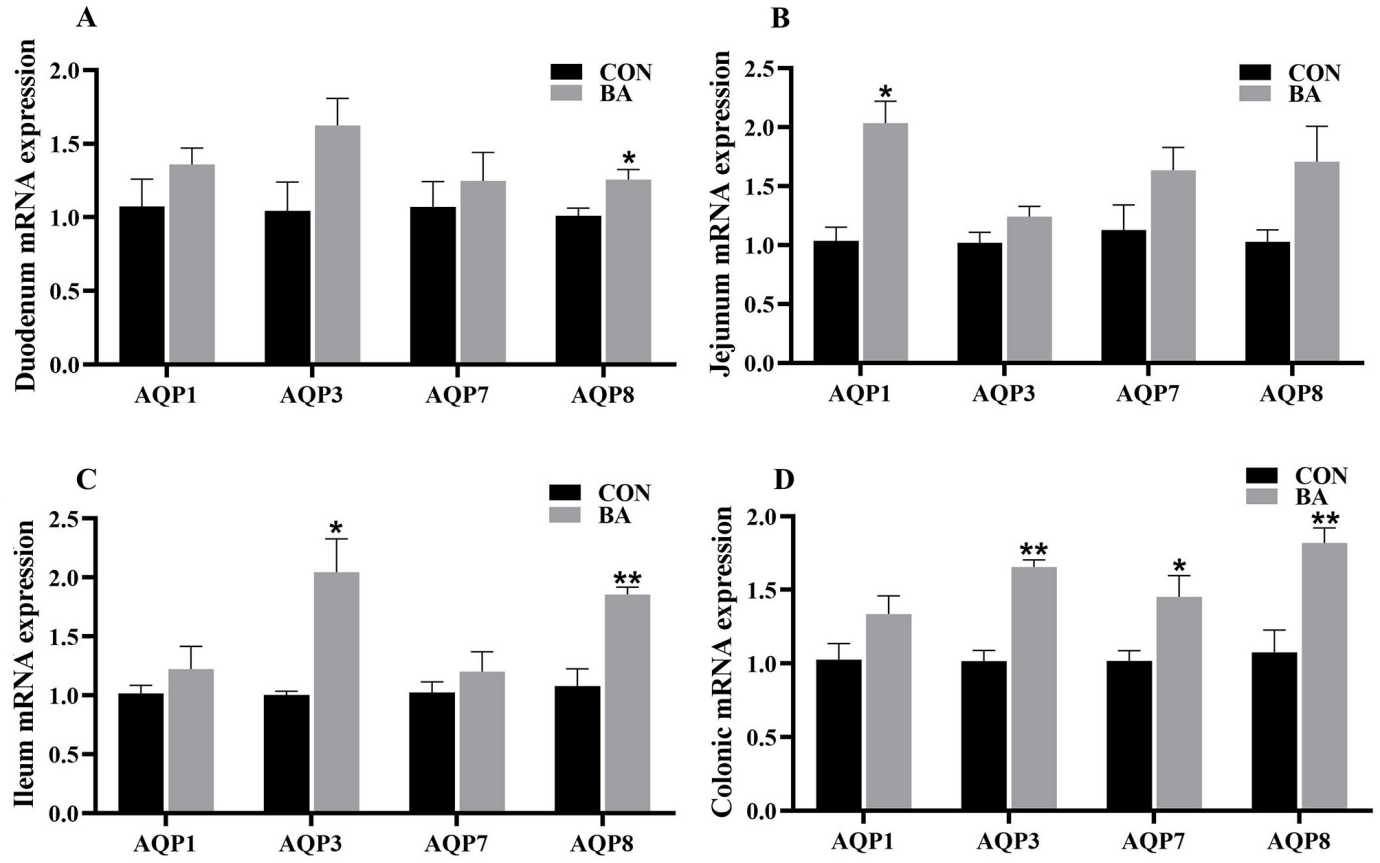
The relative mRNA expression of AQPs in the intestinal mucosa of piglets. AQPs, aquaporins. **A**, duodenum; **B**, jejunum; **C**, ileum; **D**, colon. *n* = 8. *,0.01 < *P* ≤ 0.05. **, *P* ≤ 0.01

In the duodenum (Fig. 7A), the relative mRNA expression of AQP8 in the BA group was significantly higher than that in the CON group (*P* < 0.05). In the jejunum (Fig. 7B), the relative mRNA expression of AQP1 in the BA group was significantly higher than that in the CON group (*P* < 0.05). In the ileum (Fig. 7C), the relative mRNA expression of AQP3 and AQP8 in the BA group were significantly higher than those in the CON group (*P* < 0.05). In the colon (Fig. 7D), the relative mRNA expression of AQP3, AQP7, and AQP8 were significantly higher than those of the CON group (*P* < 0.05).

### Microbes in the ileal contents of piglets

**Fig. 8:**
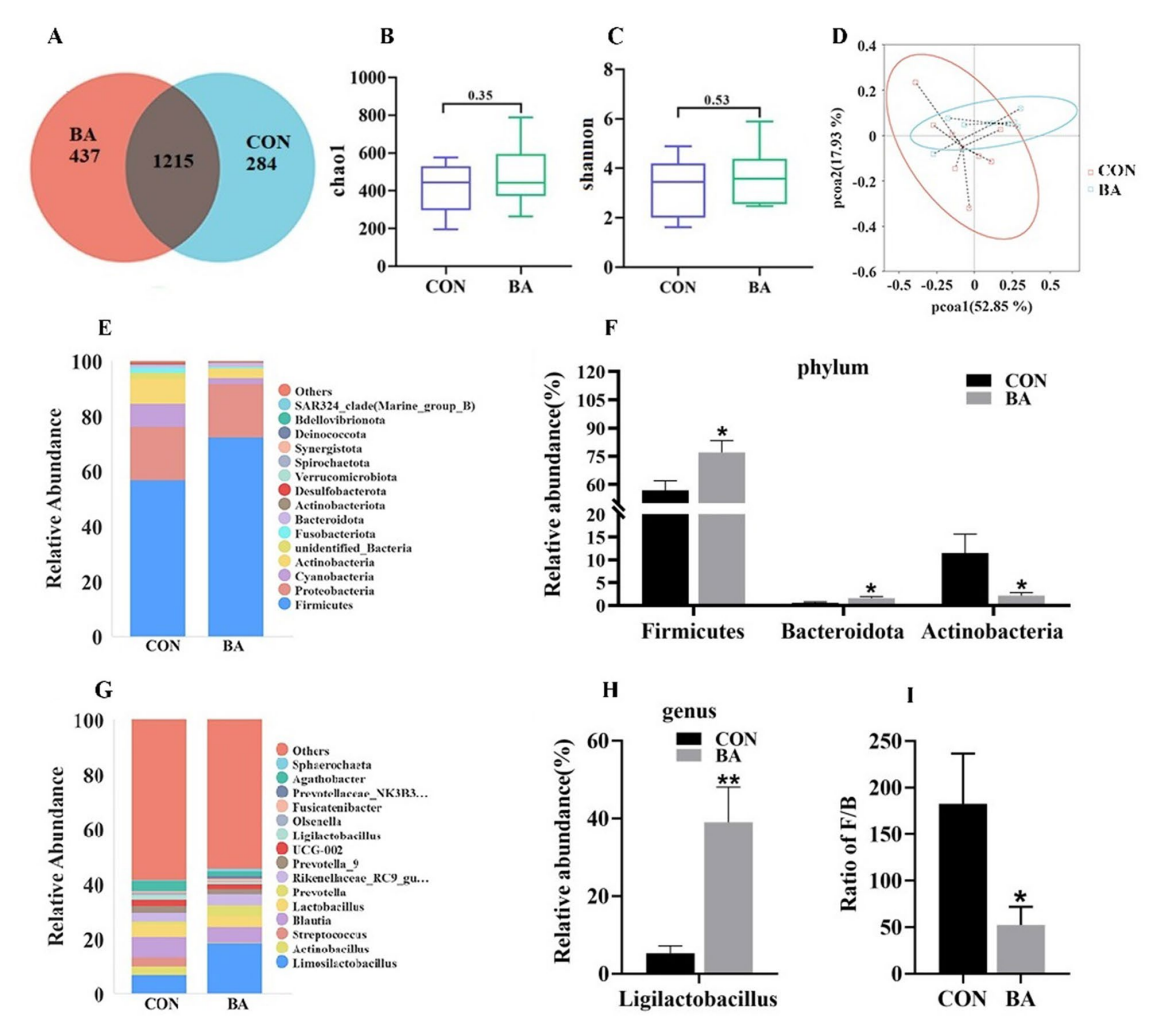
Effects of dietary bile acid supplementation on microbial composition of the ileal microbiota of weaned piglets. **A**, Venn plot. **B**-**C**, Alpha diversity. **D**, PCoA score plot. **E**-**H**, Relative abundance of microbiota in the CON and BA groups at phylum and genus levels. I, The ratio of Firmicutes and Bacteroidetes(F/B). Values are presented as the mean ± SEM (*n* = 8). *, 0.01 < *P* ≤ 0.05. **, *P* ≤ 0.01, versus the CON group

The microbial analysis results of piglet ileum contents are shown in Fig. 8. The Venn picture presented 1215 shared OTUs between two groups, and there were 437 and 284 unique OTUs in the CON and BA groups, respectively (Fig. 8A). The alpha diversity of the ileal microbiota indicated by Chao1 and Shannon (Fig. 8B-C). The analysis results showed that there was no significant difference between the CON group and the BA group. For beta diversity, the PCoA analyses showed that the microbiota in the BA group obviously tended to separate from the CON group (Fig. 8D). The major bacterial phyla in the ileum of piglets are Firmicutes (56.92% or 72.54%), Proteobacteria (19.46% or 19.29%), Cyanobacteria (8.24% or 2.31%), and Actinobacteria (9.15% or 3.22%), accounting for over 93% of the total bacterial community. The relative abundance of Firmicutes in the BA group increased from 56.92 to 72.54% compared with CON group (Fig. 8E-G). Meanwhile, compared with the CON group, the relative abundances of Cyanobacteria and Actinobacteria decreased from 8.24%, 9.15–2.31%, 3.22%, respectively. A major source of changes in genus level is *Ligilactobacillus*, which increased from 5 to 39% (Fig. 8F-H).

**Fig. 9:**
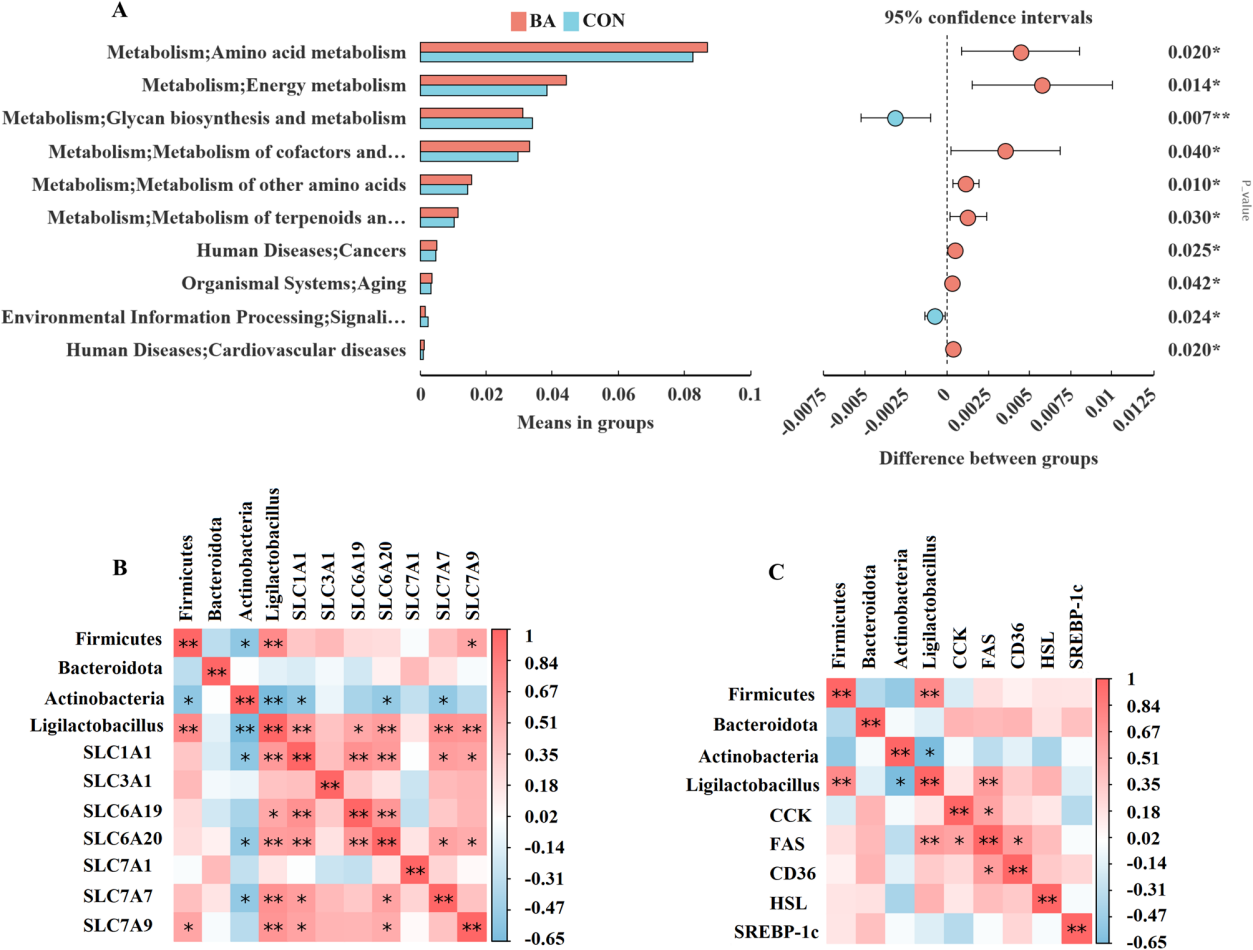
Tax4Fun analysis of microbiota in the ileum of weaned piglets. **A**, Tax4Fun analysis differential enrichment of bacterial function between CON and BA groups based on the T-test test. **B**-**C**, Spearman correlation between the gut microbiota and amino acid transporters or lipase mRNA expression in ileum. Values are presented as the mean ± SEM (*n* = 8). *, 0.01 < *P* ≤ 0.05. **, *P* ≤ 0.01, versus the CON group

Tax4Fun analysis of the predicted microbial function show that BA supplementation significantly increased ileum intestinal microbiota Amino_acid_metabolism, Energy_metabolism, Metabolism_of_cofactors_and_vitamins, Metabolism_of_other_amino_acids, Metabolism_of_terpenoids_and_polyketides and Excretory_system, significantly decreased Enzyme_families and Immune_diseases (Fig. 9A) (*P* < 0.05). In addition, the gene expression levels of amino acid transporters related genes (SLC7A9) were significantly positively correlated with the abundance of Firmicutes, the gene expression levels of amino acid transporters related genes (SLC1A1, SLC9A9, SLC6A20, SLC7A7 and SLC7A9) were significantly positively correlated with the abundance of *Ligilactobacillus*, and the gene expression levels of amino acid transporters related genes (SLC7A1, SLC6A20 and SLC7A7) were negatively positively correlated with the abundance of *Actinobacteria* in ileum (*P* < 0.05) (Fig. 9B). The gene expression levels of lipase related genes *FAS* were significantly positively correlated with the abundance of *Ligilactobacillus* (Fig. 9C).

### Effects of CDCA on the damage repair ability of IPEC-J2 cells

**Fig. 10:**
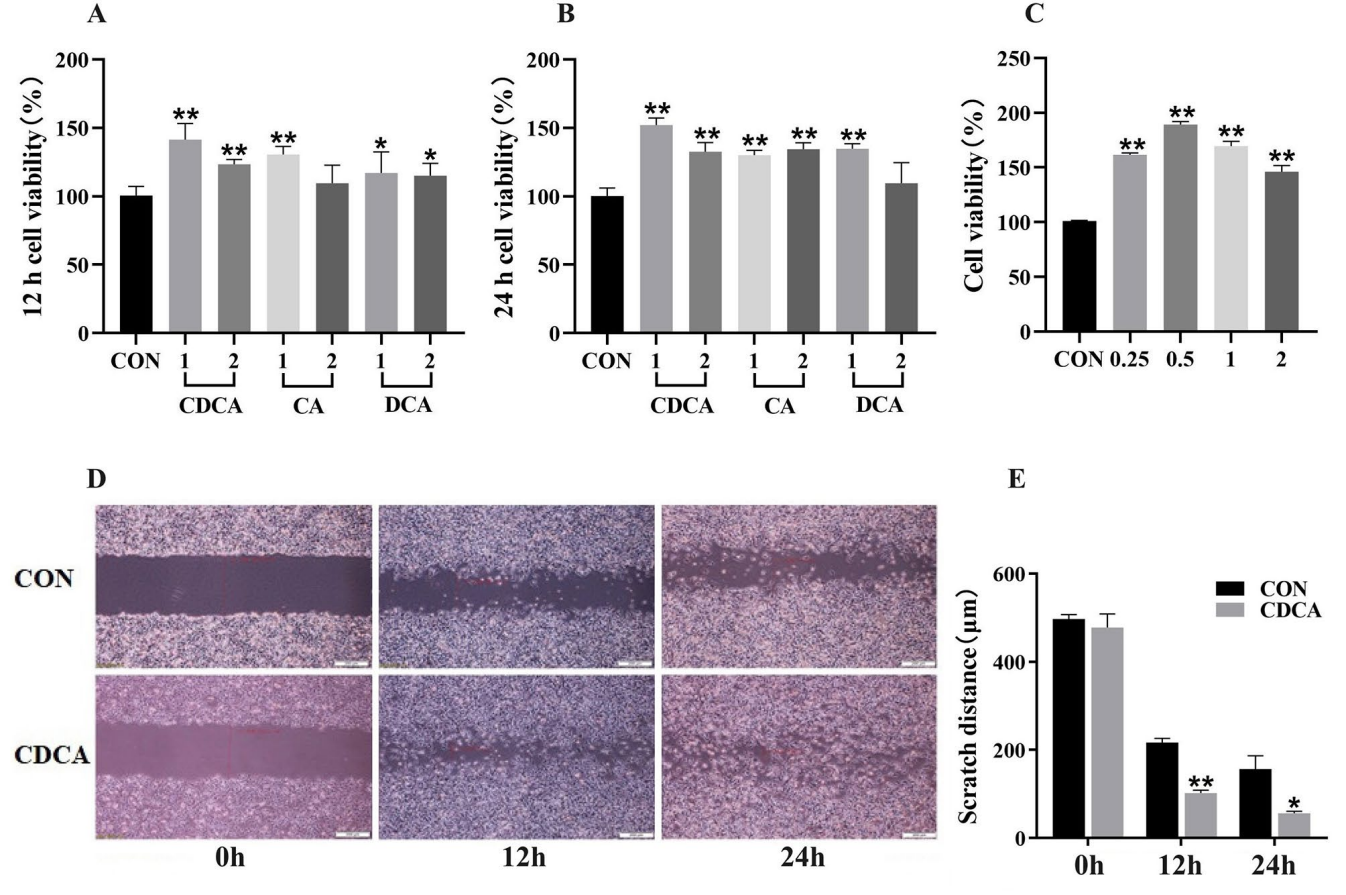
Effects of CDCA on the viability and damage repair ability of IPEC-J2 cells. **A**-**B**, Effects of different bile acid monomers on the viability of IPEC-J2 cells. CDCA, chenodeoxycholic acid; IPEC-J2, porcine small intestine epithelial cell model. **C**, Effects of different concentrations of CDCA on the viability of IPEC-J2 cells. **D**-**E**, Cell scratch mapping and scratch distance quantification. *n* = 6. *, 0.01 < *P* ≤ 0.05. **, *P* ≤ 0.01

In order to investigate the role of BA in improving the damage repair ability of intestinal epithelial cells (IPEC-J2 cells), a cell scratch test was designed firstly in this study (Fig. 10). Compared with the CON group, the addition of 0.5 µmol/L CDCA, deoxycholic acid, and cholic acid to the culture medium significantly increased the viability of IPEC-J2 cells at 12 h and 24 h (*P* < 0.05). Furthermore, the results showed that 0.5 µmol/L CDCA had the best effect on improving cell viability among all treatment groups (Fig. 10A-C). Therefore, 0.5 µmol/L CDCA was selected as a representative substance to continue the later experiments. The results of scratch test showed that 0.5 µmol/L CDCA significantly reduced the scratch distance of IPEC-J2 cells at 24 h and 48 h compared with the CON group (Fig. 10D-E) (*P* < 0.05).

### The role of CDCA in alleviating intestinal barrier impairment induced by ETEC K88

**Fig. 11:**
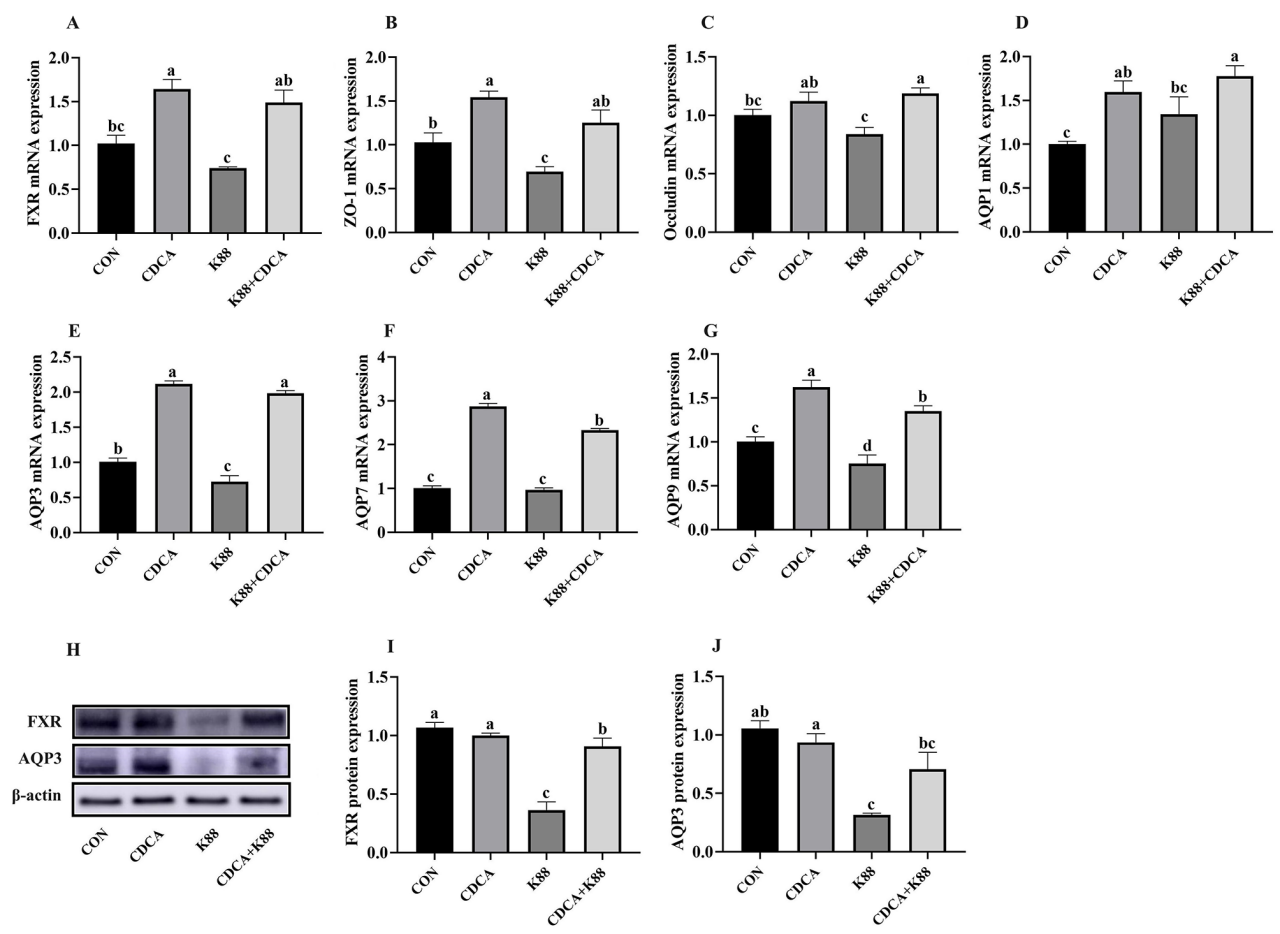
Effects of CDCA on ETEC K88 induced epithelial barrier impairment in IPEC-J2 cells. FXR, farnesoid X receptors; ETEC K88, Enterotoxigenic *Escherichia coli* (ETEC) K88. **A**-**G**, FXR, tight junction and AQPs mRNA expression in IPEC-J2 cells. H-J, FXR and AQP3 protein expression in IPEC-J2 cells. *n* = 6. a, b, c, d, *P* < 0.05

**Fig. 12:**
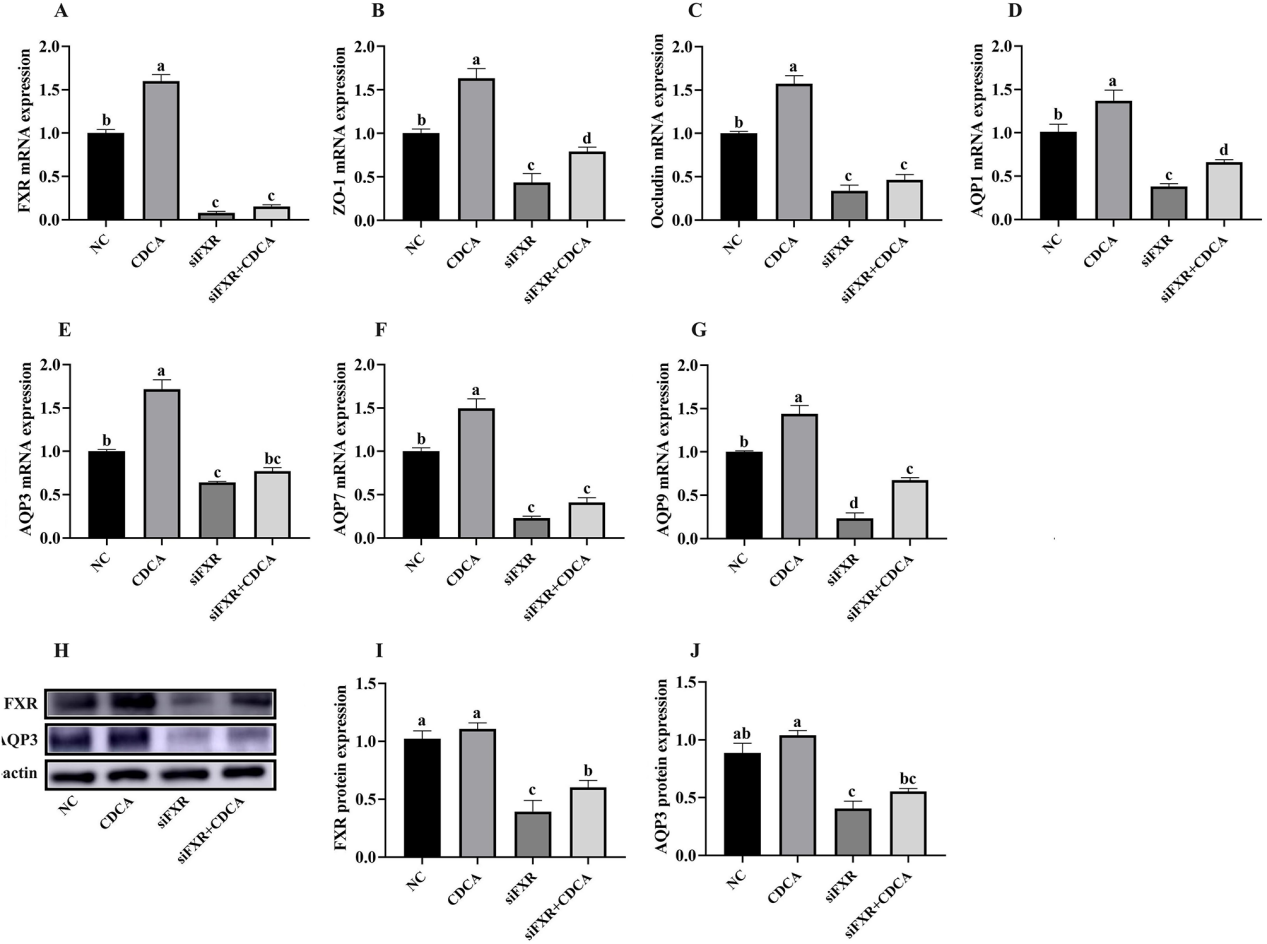
The effect of FXR siRNA interference on the expression of key genes related to intestinal barrier function. A-G, the mRNA expression of FXR, tight junctions and AQPs in IPEC-J2 cells. H-J, the relative protein abundance of FXR and AQP3 in IPEC-J2 cells. *n* = 6. a, b, c, d, *P* < 0.05

In order to investigate the mechanism of CDCA in alleviating intestinal barrier damage, this study designed a cellular inflammatory model induced by ETEC K88 (Figs. 11 and 12). As shown in Fig. 11, compared with the CON group, CDCA supplementation significantly increased the relative mRNA expressions of FXR, ZO-1, AQP1, AQP3, AQP7 and AQP9 in IPEC-J2 cells (*P* < 0.05). Moreover, CDCA enhanced FXR, ZO-1, Occludin, AQP1, AQP3, AQP7 and AQP9 mRNA expression in IPEC-J2 cells (*P* < 0.05), and thereby alleviated ETEC K88 induced intestinal barrier impairment. Subsequently, this study suppressed the gene expression of FXR through siRNA interference to verify its role in regulating intestinal barrier function (Fig. 12). The results showed that CDCA supplementation significantly increased the relative mRNA expression of FXR, ZO-1, AQP1, AQP3, AQP7 and AQP in IPEC-J2 cells compared with the CON group (*P* < 0.05). Compared with the control group, FXR siRNA interference significantly reduced the mRNA expression of FXR, ZO-1, Occludin, AQP1, AQP3, AQP7, and AQP9 in IPEC-J2 cells.

## Discussion

Gastrointestinal tract of early weaned piglets is highly susceptible to functional damage caused by external stimuli due to incomplete development, which ultimately damages their growth potential [2, 3]. This study found that dietary bile acid supplementation improved the growth performance, and reduced F/G and diarrhea rates of piglets, but there was no significant difference in feed intake. These results indicated that bile acid may improve the growth performance of piglets by increasing nutrient digestibility or/and promoting nutrient absorption in the intestine. Weaning can lead to insufficient secretion of bile acid and lipase, thereby reducing the digestibility of dietary fat and limiting the development of growth potential in weaned piglets [22]. CDCA is a main primary bile acid that naturally exists in human and animal bile. Song et al. (2021) reported that dietary supplemented with 200 mg/kg CDCA significantly improved the final weight, ADG, and feed efficiency of weaned piglets, as well as a trend towards reducing diarrhea. The enhancement of pancreatic lipase secretion and activity with the help of bile acid is an important factor in improving nutrient digestibility [18]. However, another study reported that dietary supplemented with 60 mg/kg CDCA showed no significant difference in ADG, ADFI, and Feed/Gain ratio of weaned piglets [19]. Thus, in order to explain the mechanism by which bile acids improve the growth performance and diarrhea of piglets, this study investigated the absorption of nutrients in the intestine and the intestinal barrier function.

An undamaged intestine is beneficial for weaned piglets to absorb and utilize nutrients [23]. This study found that dietary supplementation with bile acid reduced the crypt depth of intestine, increased the ratio of villus height to crypt depth, which is beneficial for improving the absorption efficiency of nutrient. Similarly, Song et al. (2021) reported that dietary supplementation with CDCA increased the ratio of villus height to crypt depth and the number of goblet cells in the intestinal mucosa of piglets, suggesting that bile acid can improve the morphology and function of piglet intestines. Importantly, as mentioned earlier that dietary supplementation with CDCA promoted lipid metabolism [18]. The results of this study showed that dietary bile acid supplementation increased the content of lipase in serum and reduced the retention of total cholesterol, triglyceride, and LDL in the serum. Furthermore, a significant increase in the mRNA expression of key genes related to lipid metabolism in the intestinal mucosa and liver of piglets, and the mRNA expression of partial amino acid transporters significantly increased. Therefore, dietary supplementation with bile acid showed a positive effect on improving lipid metabolism and amino acid absorption.

A healthy gut is a prerequisite for ensuring normal intestinal function. Intestinal integrity is an important indicator for evaluating the intestinal health of piglets [24, 25]. Tight junctions, as the first physical barrier, play a crucial role in resisting the invasion of external pathogenic factors and maintaining gut health. Tight junctions are mainly composed of Claudin 1, Occludin, and ZO-1. Also, AQPs is an important part of the intestinal mucosal barrier, and the low expression of AQPs can lead to the increase of intestinal mucosal permeability [26]. Previous study reported that dietary supplementation with bile acid (such as CDCA) reduced intestinal permeability in weaned piglets [19]. Song et al. (2021) reported that dietary supplementation with CDCA improved the expression of tight junction proteins. In addition, a study conducted on mice showed that CDCA can alleviate LPS induced damage to tight junction proteins and permeability in the intestine [16]. Similarly, this study found that dietary supplementation with bile acid improved the expressions of tight junctions and AQP proteins in the intestine. Therefore, dietary supplementation with bile acid is beneficial for improving the integrity of piglets’ intestines.

Gut microbiota is not only an important factor affecting the digestion and absorption of nutrients, but also a component of the intestinal barrier. Increasing evidences suggested that a close correlation between bile acid and gut microbiota [14]. This study showed that dietary supplementation with bile acid increased the relative abundance of Firmicutes, which is mainly related to the increase in relative abundance of Ligilactobacillus. Ligilactobacillus (*Ligilactobacillus salivarius*) has been gaining attention as a promising probiotic. The functional characteristics exhibited by *Lactobacillus salivarius* are sometimes shared with other members of the *Lactobacillus salivarius* branch and other lactobacilli, such as *Lactobacillus reuteri*, *Lactobacillus casei*, and *Lactobacillus rhamnosus* GG [27]. Numerous strains of *Lactobacillus* exhibited functional characteristics with health benefits, such as antibacterial activity, immunological effects, and the ability to regulate the gut microbiota [28]. In an early study, *Lactobacillus salivarius* was reported to enhance host immunity by inducing IL-10 and some immunoglobulin levels, as well as inducing an increase in the number of monocytes and natural killer cells [29]. Recent research has also shown that certain strains of *Lactobacillus salivarius* have antibacterial activity in animals, which can prevent the colonization of *Salmonella enteritidis* in chickens and reduce and prevent intestinal pathogen infections in piglets and calves [30–33]. Therefore, dietary bile acids supplementation may improve intestinal function in piglets by increasing the abundance of probiotics, such as Lactobacillus.

Moreover, as a signaling molecule, bile acid can regulate various metabolic activities by activating bile acid receptors FXR [15, 16, 34]. FXR played an important role in regulating intestinal permeability or barrier integrity [34]. The activation of FXR reduced the production of pro-inflammatory cytokines, thereby contributing to a decrease in epithelial permeability [35]. On the contrary, it has been shown that the intestinal epithelial barrier of FXR knockout mice is weakened [36]. Therefore, this study further explored the potential role of FXR/AQPs pathway in bile acids-alleviating intestinal barrier impairment induced by ETEC K88. Firstly, this study compared the effects of different concentrations of CDCA, deoxycholic acid and cholic acid on the cell viability of IPEC cells, and the results showed that CDCA had the best effect on the improvement of IPEC-J2 cell viability. Therefore, CDCA, as a representative component of bile acids, was selected for subsequent regulatory mechanism studies in this study. The results of this study indicated that CDCA showed a positive role in improving the expression of FXR, AQPs, and tight junctions. Moreover, after being challenged by ETEC K88 or interfered with FXR siRNA, the expressions of AQPs and tight junctions were significantly inhibited. However, this study found that CDCA can improve the negative effects of ETEC K88 attack or FXR siRNA interference on the expression of AQPs and tight junctions in IPEC-J2 cells. These results indicated that bile acid may partially regulate the expression of AQPs and tight junctions through FXR. Previous studies have shown that changes in intestinal AQPs expression have been proven to be associated with diarrhea [37, 38]. An early study reported that infection with pathogenic *Escherichia coli* and hemorrhagic *Escherichia coli* can cause diarrhea in mice, and revealed that this is related to the change of AQP localization in colonocytes [39]. Song et al. (2019) showed that CDCA mainly improves epithelial barrier impairment in IPEC-J2 cells through FXR, rather than TGR5. Therefore, there may be other pathways for bile acid to regulate the expression of AQPs and tight junctions.

## Conclusion

**Fig. 13:**
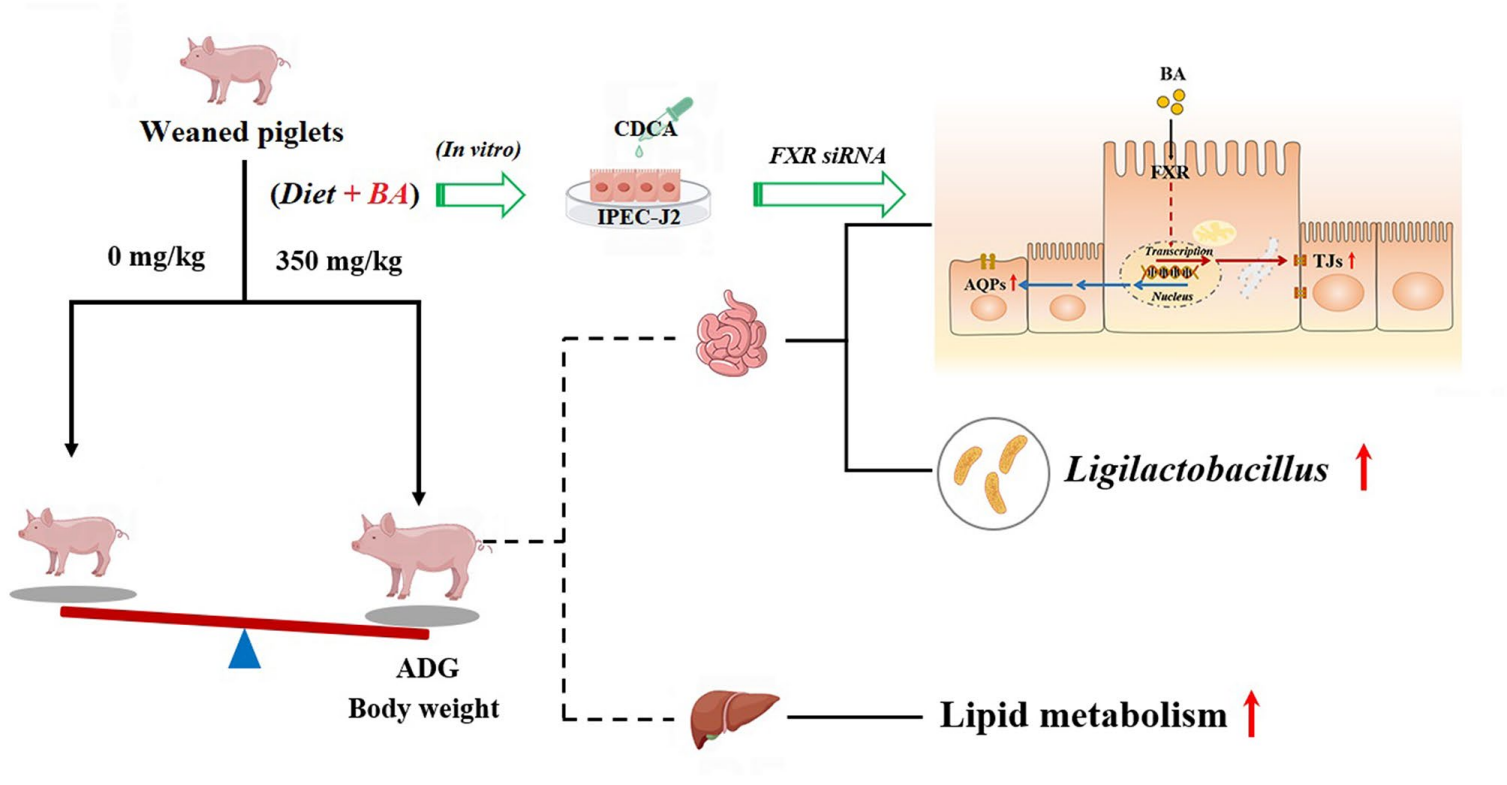
Schematic diagram of experimental procedure to study the effects of bile acid on growth performance and intestinal health of weaned piglets

In summary, dietary supplementation with bile acid is beneficial for improving the growth performance and intestinal health of weaned piglets, which is related to the improvement of intestinal morphology, amino acid transport and lipid absorption in the intestine, the expression of tight junction proteins and aquaporins, and increased abundance of Lactobacilli by bile acids (Fig. 13). Meanwhile, this study found that bile acid improved the repair of IPEC-J2 cells after injury and was demonstrated to be associated with the activation of farnesol X receptor (FXR) and increased expression of tight junction proteins and AQPs proteins.

## Electronic supplementary material

Below is the link to the electronic supplementary material.

Supplementary Material 1

## Data Availability

No datasets were generated or analysed during the current study.
