# Supplementary material for: Dietary bile acid supplementation improves the intestinal health and growth performance of piglets partially through the FXR/AQPs pathway
Source: Porcine Health Manag. 2025 May 21;11:28. doi: 10.1186/s40813-025-00440-x (PMC12093730; doi:10.1186/s40813-025-00440-x)
Supplement: Supplementary file 1 — Supplementary Material 1 [file 40813_2025_440_MOESM1_ESM.docx]

**Table S1** Primer sequences used in the study

| Genes | | Accession no. | Sequence（5'→3'） | Size |
| --- | --- | --- | --- | --- |
| BA receptor | *FXR* | NM_001287412.1 | CCGAGAGGCAGTAGAGAA | 144 |
|  |  |  | GCGTGGTGATGGTTGAA |  |
| Tight junctions | *ZO-1* | [XM_021098896.1](https://www.ncbi.nlm.nih.gov/entrez/viewer.fcgi?db=nucleotide&id=1191808335) | AGCCCGAGGCGTGTTT | 147 |
|  |  |  | GGTGGGAGGATGCTGTTG |  |
|  | *Occludin* | [XM_005666863.2](https://www.ncbi.nlm.nih.gov/entrez/viewer.fcgi?db=nucleotide&id=1191901830) | GACTCCTTGCTGAATCTGA | 139 |
|  |  |  | GCACCTCATCATCTTCCAT |  |
| Aquaporins | *AQP1* | [XM_021078524.1](https://www.ncbi.nlm.nih.gov/entrez/viewer.fcgi?db=nucleotide&id=1191850904) | CATCATTGCCCAGTGTGTGG | 145 |
|  |  |  | GCCAATGATCTCGATGCCCA |  |
|  | *AQP3* | [NM_001110172.1](https://www.ncbi.nlm.nih.gov/entrez/viewer.fcgi?db=nucleotide&id=158819035) | TGACCTTCGCTATGTGCTTCC | 212 |
|  |  |  | GTCCAAGTGTCCAGAGGGGTAG |  |
|  | *AQP7* | [XM_021063972.1](https://www.ncbi.nlm.nih.gov/entrez/viewer.fcgi?db=nucleotide&id=1191813214) | CCCGTGCCTCCAAGATGA | 58 |
|  |  |  | CGCATTATTGTTTGCATCTTTGA |  |
|  | *AQP9* | [NM_001112684.1](https://www.ncbi.nlm.nih.gov/entrez/viewer.fcgi?db=nucleotide&id=162951798) | TGTCATTGGCCTCCTGATTG | 62 |
|  |  |  | TGGCACAGCCACTGTTCATC |  |
| Fat metabolism genes | *HSL* | HM591297 | GCAGCATCTTCTTCCGCACA | 195 |
|  |  |  | AGCCCTTGCGTAGAGTGACA |  |
|  | *ATGL* | EU047807 | GCGAAAATGTCATCATAACC | 175 |
|  |  |  | ATGGTGCTCTTGAGTTCGT |  |
|  | *SREBP-1c* | NM_214157.1 | AAGCGGACGGCTCACAA | 121 |
|  |  |  | GCAAGACGGCGGATTTATT |  |
|  | *FADS2* | [NM_001171750.1](https://www.ncbi.nlm.nih.gov/entrez/viewer.fcgi?db=nucleotide&id=284519720) | GGCGCAGATGCCTACCTTTA | 148 |
|  |  |  | TGCATAGTGCGAGATGACCC |  |
|  | *FAS* | NM_001099930.1 | CCTGGGAAGAGTGTAAGCA | 108 |
|  |  |  | GGAACTCGGACATAGCG |  |
|  | *CD36* | NM_001044622 | TGTGGATACTTGGAGGTGGG | 111 |
|  |  |  | TGCTGGTTGGAATACAGTGG |  |
| Amino acid transporters | *SLC1A1* | NM_001164649.1 | AGTGAGCCAGAGACGAATGG | 73 |
|  |  |  | AAACAATCAAGCCCAGGACA |  |
|  | *SLC3A1* | NM_001123042.1 | TACCACGACTTCACCACCAC | 146 |
|  |  |  | CTCTCTCCTTGGGCTTCAGT |  |
|  | *SLC6A19* | XM_003359855.4 | TCATCTTCCTCTTCTTCTTCGTG | 155 |
|  |  |  | CTTGACCTTCTGGGATTTGG |  |
|  | *SLC6A20* | NM_020208.3 | TGGTGGTGTCCTTCTTCCTC | 134 |
|  |  |  | GTCATAGCCCGTGTGGTTG |  |
|  | *SLC7A1* | NM_001012613 | TCTGGTCCTGGGCTTCATAA | 192 |
|  |  |  | ACCTTCGTGGCATTGTTCAG |  |
|  | *SLC7A7* | NM_001110421.1 | GAGTGCCAGAACACAAACGA | 216 |
|  |  |  | TCCTCCATCTTCCAAATCCA |  |
|  | *SLC7A9* | NM_001110171.1 | GCCTATCAAGGTGCCCATC | 144 |
|  |  |  | AGCGGACGAACAGGAAGTAA |  |
|  | *β-actin* | [XM_021086047.1](https://www.ncbi.nlm.nih.gov/entrez/viewer.fcgi?db=nucleotide&id=1191864134) | TCAGCAAGCAGGAGTACGAC | 210 |
|  |  |  | TCACAGCTTCTCAGCAGACAG |  |
